# Digital Technology in Somatic and Gene Therapy Trials of Pediatric Patients With Ocular Diseases: Protocol for a Scoping Review

**DOI:** 10.2196/10705

**Published:** 2019-02-07

**Authors:** Edward Meinert, Abrar Alturkistani, Tasnime Osama, Celine-Lea Halioua-Haubold, Josip Car, Azeem Majeed, Glenn Wells, Robert E MacLaren, David Brindley

**Affiliations:** 1 Global Digital Health Unit Department of Primary Care and Public Health, School of Public Health Imperial College London London United Kingdom; 2 Healthcare Translation Research Group Department of Paediatrics University of Oxford Oxford United Kingdom; 3 Clinical Ophthalmology Research Group Nuffield Department of Clinical Neurosciences University of Oxford Oxford United Kingdom; 4 Department of Primary Care and Public Health School of Public Health Imperial College London London United Kingdom; 5 Oxford Academic Health Science Centre Oxford United Kingdom; 6 Healthcare Translation Group Department of Paediatrics University of Oxford Oxford United Kingdom

**Keywords:** clinical trial, health care, genomics, gene therapy, personalized medicine

## Abstract

**Background:**

Pharmacogenomics suggests that diseases with similar symptomatic presentations often have varying genetic causes, affecting an individual patient’s response to a specific therapeutic strategy. Gene therapies and somatic cell therapies offer unique therapeutic pathways for ocular diseases and often depend on increased understanding of the genotype-phenotype relationship in disease presentation and progression. While demand for personalized medicine is increasing and the required molecular tools are available, its adoption within pediatric ophthalmology remains to be maximized in the postgenomic era.

**Objective:**

The objective of our study was to address the individual hurdles encountered in the field of genomic-related clinical trials and facilitate the uptake of personalized medicine, we propose to conduct a review that will examine and identify the digital technologies used to facilitate data analysis in somatic and gene therapy trials in pediatric patients with ocular diseases.

**Methods:**

This paper aims to present an outline for Healthcare Information Technology and Information and Communication Technology resources used in somatic and gene therapy clinical trials in children with ocular diseases. This review will enable authors to identify challenges and provide recommendations, facilitating the uptake of genetic and somatic therapies as therapeutic tools in pediatric ophthalmology. The review will also determine whether conducting a systematic review will be beneficial.

**Results:**

Database searches will be initiated in September 2018. We expect to complete the review in December 2019.

**Conclusions:**

Based on review findings, the authors will summarize methods used for facilitating IT integration in personalized medicine. Additionally, it will identify further research gaps and determine whether conducting further reviews will be beneficial.

**International Registered Report Identifier (IRRID):**

PRR1-10.2196/10705

## Introduction

“Personalized medicine” and more specifically, genomics, is transforming health care through the implementation of strategies aiming to individualize prevention and treatment [1]. By matching patients to specific drugs and therapies, thereby providing customized and tailored treatments, patient needs, values, and preferences are considered [2]. While personalized medicine requires quantitative assessment methods including, but not limited to, genotype sequencing, it also relies heavily on the involvement of patients in their management and treatment [2]. Multiple gene therapy trials targeting corneal and retinal disorders are currently underway [3]. Ideally, retinal gene therapies will be administered to patients before significant vision loss. Gene therapy can, currently, only prevent further degeneration of retinal cells by delivering the critical missing gene, not halting disease progression or blindness [4]. Therefore, pediatric patients stand to benefit greatly from the development of gene therapies for retinal diseases. Patient-centered personalized medicine has been facilitated by the integration of informatics within health care (Healthcare Information Technology [HIT]), as well as access to information by means of telecommunications (Information and Communication Technology [ICT]), which have enabled interactions and exchange of information [5,6,7]. Developed by researchers at the Picker Institute in 1988, “patient-centered care” represents a health care delivery methodology driven by patient needs and perspectives, empowering patients to become partners in the management of their own health [8], thereby improving the quality of service delivery. In fact, according to the “Crossing the Quality Chasm” report developed by the Institute of Medicine, patient-centered care represents one of the 6 essential elements of high-quality care [9] and has the potential to shift medical practice away from the more conventional approach of “one size fits all” [10].

HIT and ICT have tremendous potential to contribute to and enhance patient-centered approaches [11]. Electronic health (eHealth), or the use of ICT in health care [12], has facilitated the flow of information and improved health care quality [8]. Although eHealth remains underutilized, its role in personalized medicine and the improvement of communication in clinical settings is significant [13]. While health systems implementing patient-centered approaches facilitate advances in personalized medicine, research suggests that promoting the utilization and integration of big data and ICT solutions in personalized medicine remains a challenge to overcome [14]. HIT, an enabling and fundamental component of health systems, facilitates the improvement of health system quality and efficiency, as well as patient safety by addressing patient needs and preferences in the right setting at the right time, subsequently enforcing active patient engagement and autonomy [15,16]. Through digital methods utilizing personal health records, or digital collection of health-related information recorded and maintained by patients [17], patients and their caregivers have additional control over their illnesses [18,19], improving patient engagement [20]. The wide adoption of HIT requires addressing issues including data storage, fragmentation of data, and lack of interoperability to be addressed before the full potential of this strategy may be realized in personalized medicine.

Recent advances in scientific technology, including facile genome sequencing, advanced genome editing techniques, and controlled isolation and differentiation of cells *in vitro*, have clear translational value [21]. In addition to understanding why individuals with similar diseases respond differently to different therapeutics, big data in health care can allow researchers to determine why individuals with analogous conditions are attributed different prognoses [14], enabling early intervention through risk stratification [22]. Therapies that utilize these and related strategies are referred to as “gene therapy medicinal product” and “somatic cell therapy medicinal product” by the European Medicines Agency [23]; here we will refer to them as gene therapy and somatic cell therapy, respectively. Somatic cell therapy refers to the administration of processed or manipulated somatic cells to alter biologic characteristics [24]. Gene therapy alters genes in targeted cells, thereby preventing and treating disorders [24]. “Corrected” versions of the gene may be delivered to the patient’s cells [25], for example by adeno-associated virus, ameliorating disease prognosis. These therapeutic strategies may be combined by first genetically modifying cells *ex vivo* before delivering them to the patients [22]. As inherited diseases are clearly amenable to a gene therapy strategy, pediatric patients represent a population that may gain tremendously from advances in this field [3].

New technologies in health care are allowing physicians to tailor and customize treatments to individual patients, translating genomic research into medical practice. Acting as a driving force, novel technologies are central to the advancement of gene and somatic therapy trials. As there is no existing synthesis of the electronic processes used in somatic and gene therapy trials in pediatric patients with ocular diseases, this gap will be addressed by the proposed review.

## Methods

### Background

We will use the Preferred Reporting Items for Systematic Reviews and Meta-Analyses Protocol PRISMA-P 2015 Checklist (Multimedia Appendix 1) and the Cochrane protocol guide to develop the protocol. In order to establish Medical Subject Headings (MeSH), subject heading, and keywords (Multimedia Appendix 2), a clear research question will be developed and a Population, Intervention, Comparator, and Outcome framework will be completed. To provide replicable and transparent methods, we will describe the following 6 stages in detail: (1) Literature search; (2) article selection; (3) data extraction; (4) quality appraisal; (5) data analysis; and (6) data synthesis.

Systematic scoping reviews intend to promptly gather and provide evidence on essential concepts pertaining to broad research subjects [26]. As opposed to systematic reviews that answer narrow and specific questions, scoping reviews may answer broad questions and provide evidence that lie outside of the effectiveness of an intervention [25]. Thus, where systematic reviews may not be conducted due to evidence deficiency, systematic scoping reviews have been proven to be valuable [27].

Although scoping reviews do not require a strict methodological approach, methodological frameworks have been developed by Arksey and O’Malley in 2005 and by Levac, Colquhoun, and O’Brien in 2010 [26,27].

### Identification of a Research Question

Following preliminary and exploratory reading on somatic and gene therapy trials involving pediatric patients with ophthalmic conditions, no synthesis of evidence around the technological advances used in these trials was found, demonstrating a research gap. In light of this evidence paucity, we formulated the following research question: What are the range of health care information and communication technologies applied to achieve patient-centered care in children undergoing somatic and gene therapies for ocular diseases?

### Search Strategy

We will systematically searche MEDLINE/PubMed, Embase, and Scopus through the Imperial College London library. Subsequent to the identification of MeSH, subject headings, and keywords, the medical librarian at Imperial College London will be asked to review the search strategy. Search terms will consist of MeSH and keywords linked to (1) pediatric population; (2) digital technologies; (3) traditional care; and (4) outcomes. No restrictions will be applied on publication date, publication status, or study location. Subsequent to the MEDLINE/PubMed search, the search strategy will be converted to Embase and Scopus searches. The search strategy may be requested from the first author once finalized. Following database searches, results will be imported to EndNote, a reference software. Once the included studies are determined, we will thoroughly search their bibliographic citations to identify supplemental relevant studies.

### Inclusion and Exclusion Criteria

The following Population, Intervention, Comparator, and Outcome framework was developed for the purpose of the review’s research question.

#### Population

Eligible studies will consist of pediatric patients with ocular diseases undergoing somatic and gene therapy trials.

#### Intervention

Interventions will consist of advances in health care technologies, including, eHealth, mobile health, big data, and real-world data utilized in genomic and somatic therapies in children with ocular diseases.

#### Comparison

No comparisons will be made.

#### Outcomes

Treatment and process outcomes will be assessed. Treatment outcomes will consist of clinical effectiveness, clinical efficacy, and patient-relevant outcomes. Process outcomes will evaluate the quality and accessibility of technological advances.

#### Study Type

The following studies will be included (1) No restrictions will be placed on study type; (2) English publications; (3) publications obtained from MEDLINE/PubMed, Embase, and Scopus; and (4) publications focusing on digital technologies used in pediatric somatic and gene therapy clinical trials.

#### Exclusion Criteria

We excluded non-English publications.

### Study Record Management

EndNote X8.2 (Clarivate Analytics) will be used for the collection of bibliographic references. Following the assemblage of references following database searches, duplicates will be removed using a deduplication tool on the bibliographic software. Where duplicates are not removed automatically by means of the EndNote software, reviewers will examine and compare findings thoroughly. Upon agreement, duplicates will be removed. Authors of included studies will be contacted if required.

### Study Selection

In the first phase of the selection process, 2 reviewers will independently perform screening of titles and abstracts. Following elimination of papers with discernible ineligibility, eligibility of the remaining papers will be assessed in the second phase of the study selection process through full-text reading. Disagreements on study eligibility will be resolved through discussion between reviewers. If no consensus is reached, a third reviewer will assist in the selection process. In the final phase of the selection process, bibliographic references of included studies will be reviewed, thereby identifying additional potentially relevant papers. A PRISMA flow diagram will be used to illustrate the selection process, as well as exclusion reasons, demonstrating the review’s transparency and replicability.

### Data Extraction

#### Data Extraction Process

We will extract data from included studies and compile onto data extraction forms designed by the research team. Prior to comparison of completed data extraction forms, data will be extracted independently by 2 reviewers. Differences in opinions will be resolved through discussion and, if required, assistance of a third reviewer. A single form consisting of the required data will be generated.

**Table 1:** Details of extracted data.

Type of extracted data	Details of extracted data
Paper information	Author Date of publication Country where the clinical trial was conducted
Study characteristics	Clinical trial setting Type of somatic or gene therapy Trial duration or length Sample size Use of a control Follow-up duration
Participant characteristics	Number of participants Age of participants Gender of participants
Intervention details	Type of technological advances Information technology use in somatic or gene therapy trials (stage) Challenges around implementation Enabling factors
Comparator details	Type of comparator (traditional approach, standard care, none)
Outcome measures	Treatment outcomes Process outcomes

#### Data Items

Data extracted from included studies will consist of the following information: (1) paper specifications; (2) study characteristics; (3) participant characteristics; (4) intervention details; (5) comparator details; and (6) outcome measures. Details pertaining to extracted data may be found in Table 1. Through collection of the extracted data, evidence around the technological advances used in somatic and gene therapy trials will be presented and discussed. To ensure adequate collection of data, the team will review the data extraction forms prior to usage.

### Risk of Bias Assessment of Included Studies

The methodological quality of included studies will be assessed by 2 independent reviewers. If, following discussion, reviewers cannot reach consensus on the risk of bias pertaining to included studies, a third reviewer will be asked to aid in decision making.

To assess the methodological quality of included studies, the Cochrane Collaboration Risk of Bias Tool will be applied. The following 6 criteria will be evaluated in each included study: (1) random sequence generation (selection bias); (2) allocation concealment (selection bias); (3) blinding (performance bias and detection bias); (4) incomplete outcome data (attrition bias); (5) selective reporting (reporting bias); and (6) other bias.

Following criteria assessment, risk of bias of included studies will be determined, and studies will be judged as “high risk,” “low risk,” or “unclear risk.” To illustrate the methodological quality of the review’s included studies, a Risk of Bias Graph and a Risk of Bias Summary will be presented.

### Data Synthesis

Subsequent to data extraction, results will first be synthesized numerically or by means of a descriptive statistic. Providing an overview of the quantity and type of included papers, the descriptive analysis will consist of the following information: (1) intervention type, control group, sample size; (2) characteristics of the population involved including, age, gender, ocular disease targeted, location; and (3) intervention outcomes including, health treatment and process outcomes

Results will also be synthesized narratively. Using the PRISMA-P 2015 Checklist, data will be presented in a tabular format to supplement the narrative synthesis. This will consist of a synthesis of all included papers that will enable guidance and allow assessment of potential heterogeneity between included studies. To ensure the reliability of our findings, narrative synthesis reporting will be conducted in a transparent manner.

Providing a holistic analysis of the intervention, the quality of the studied intervention will be determined by evaluating through outcome measures, as well as satisfaction of health care professionals and patients and the complexity of the intervention. Recommendations regarding future research, policy, and practice will be developed by the authors. Details of this stage are currently being developed by the research team and may be subjected to iterations or further updates following review commencement. We intend to finalize this stage in December 2017.

## Results

Database searches will be initiated in September 2018. We expect to complete the review in December 2019.

## Discussion

### Overview

The review intends to provide evidence on technology applied to genetic and somatic cell therapy trials in children with ocular diseases. In addition to developing recommendations that will enable collaboration between key stakeholders, we aim to provide a comprehensive overview that will facilitate decision making and improve pediatric health care. Thus, we aspire to move evidence into practice, or translate research into medical practice, through the circulation of our review findings. We predict that the review will also provide insight to other researchers as additional research gaps that may need to be addressed will be identified by means of evidence gathered in the proposed review.

### Strengths

Responding to a research gap, the review will provide evidence on technological advances related to somatic and gene therapy trials in children. We will perform rigorous and systematic search of multiple health and medicine databases. No restrictions applied on publication date, status, or location. The review will identify unmet needs of pediatric patients with ophthalmologic disorders, thereby informing policy makers and donors. On completion of the review, additional research gaps will be identified, thereby guiding the conduct of future systematic reviews. Review findings will enhance the management of pediatric patients undergoing somatic and gene therapies by identifying digital technologies that improve patient safety, engagement, and satisfaction. The effectiveness of digital technologies on treatment outcomes will be assessed.

### Limitations

Language restrictions will be imposed (non-English papers will be excluded). As the review will outline the existing electronic processes applied in somatic and gene therapy trials for children, quality appraisal will not be conducted on identified papers.

### Consultation

We believe consulting experts will provide valuable insight regarding the development of recommendations, challenges that need to be addressed, and potential solutions. In addition to consulting pediatric somatic and gene therapy experts and researchers, regulatory authorities and eHealth experts at Imperial College London, the University of Oxford, and Stanford University will be consulted throughout the conduction of the review. This will allow the reviewers to obtain guidance that has not been provided in the searched literature. Their feedback on the protocol and review findings will be requested prior to completion of the final report. Through multiple consultations, we intend to engage subject experts in the development and design of the systematic review in addition to the subsequent action plan. It is intended that by means of various consultations, we will develop strategies facilitating the uptake of novel technological advancements by researchers, health care practitioners, and patients in the field of personalized medicine.

### Knowledge Translation and Dissemination

The review intends to provide evidence on the digital technologies used in data analysis and interpretation in pediatric somatic and gene therapy trials. The review intends to develop and circulate strategies to promote their uptake, enhance patient safety and enhance development of personalized medicine within health care systems. In order to enable informed decision-making skills of consumers and improve health care quality and access, results will be actively communicated to health care providers, patients, and other relevant stakeholders. To ensure transparency, replicability, and applicability of the proposed systematic review, we intend to distribute our work from the initial stage (protocol) to the final product.

### Conclusions

Genomics is reshaping health systems. While advancements in technologies are enabling the personalization and customization of care, there is paucity around the evidence of their utilization and impact in somatic and gene therapy trials in children. As eHealth and mobile health resources have the potential to empower patients through enhancement of decision-making skills and patient engagement, the review intends to exhibit how their utilizations may be maximized in pediatric somatic and gene therapy trials to improve the quality of pediatric ophthalmology.

## Appendix

Multimedia Appendix 1 The PRISMA-P 2015 checklist.

Multimedia Appendix 2 MEDLINE/PubMed search strategy.

## Abbreviations

eHealth: electronic health
HIT: Healthcare Information Technology
ICT: Information and Communications Technology
MeSH: Medical Subject Headings
