# Supplementary material for: Digital Technology in Somatic and Gene Therapy Trials of Pediatric Patients With Ocular Diseases: Protocol for a Scoping Review
Source: JMIR Res Protoc. 2019 Feb 7;8(2):e10705. doi: 10.2196/10705 (PMC6383115; doi:10.2196/10705)
Supplement: Multimedia Appendix 2 [file resprot_v8i2e10705_app2.pdf]

APPENDICES

Appendix 2: Table displaying the MEDLINE/Pubmed Search Strategy

Category	MeSH	Keywords
healthcare information and communication technologies	Medical Informatics<br>Telemedicine	electronic patient record$ OR electronic medical record$ OR personal health record$ OR Health information exchange OR technology OR telemedicine or text message$ OR sms OR telephone OR computerized decision support system OR public health informatic$ OR cellular phone OR smartphone$ OR mobile$ OR ipad$ OR computer-assisted OR user-computer interface OR personal digital assistant or computer$ OR handheld or electronic wearable device$ OR electronic wearable technology OR data OR mHealth OR Big data OR eHealth
Children	Child<br>PEDIATRICS<br>Infant<br>ADOLESCENT	child$ or infant$ or pediatric or adolescent$
somatic and gene therapies	Genetic Therapy<br>Gene Transfer Techniques<br>Genetic Therapy	Somatic-Cell Therap$ OR Gene Therap$
ocular diseases	Vision, Ocular<br>Eye Diseases<br>Retinal Diseases<br>Vision Disorders<br>Ocular Motility Disorders	Cataracts OR binocular vision or accommodative dysfunction$
